# Is there evidence for the impact of poor oral health on school performance?

**DOI:** 10.1186/s12903-019-0822-5

**Published:** 2019-07-11

**Authors:** Mir Faeq Ali Quadri, Basaruddin Ahmad

**Affiliations:** 1 School of Dental Sciences, Health Campus, Universiti Sains Malaysia, Kubang Kerian, 16150 Kelantan Malaysia; 2 Department of Dental Public Health, Jazan University, Jizan, Saudi Arabia

**Keywords:** Oral health, Child, School performance, Study design, Appraisal

## Abstract

As part of our study, we reviewed the report published in BMC-Oral Health, titled *“An assessment of the impacts of child oral health in Indonesia and associations with self-esteem, school performance and perceived employability” by Maharani et.al, 2017*. We noted a plausible error in the interpretation of results in the report and re-examined the published data. Contradictory to the published report, our analysis showed no evidence for the relationship between toothache and poor school performance. Significant relationship was only found between plaque accumulation and school performance. We argued that the error may have originated from an unclear objective and misclassification of school performance variable before applying statistical test to address the objective of this study.

## Background

A report in BMC - Oral Health (Maharani et al., 2017) had suggested that there was a significant association between poor oral health condition and academic performance in a study on Indonesian school children [1]. In the report, school performance was measured using mathematics test score which was categorised as Underachievers (Score = 0–54); Fair (Score = 55–64); Good (Score = 65–79); and Excellent (Score = 80–100). Two oral health conditions were assessed: the amount of clinically assessed plaque (substantial / not substantial) and self-reported history of toothache in the past 12 months (Yes / No). Analysis was carried out separately for 6–7 and 10–11 years age groups. Table 3 in the report showed a significant *p*-values (< 0.05) from chi-square tests for the relationship between plaque and toothache and school performance. The authors stated that “*Children aged 10–11 years who had experienced toothache were found to have significantly lower school performance than their peers*” and later concluded that toothache was associated with poor school performance. We argued that the interpretation of the results in Table 3 of the report was inappropriate and aimed to demonstrate how the analysis can be improved.

Table 3 in Maharani et al. [1], showed that generally, the percentages of children with substantial plaque or a history of toothache whom had fair or good grades were greater than those without substantial plaque or toothache; but the percentages were lower in those whom had an excellent grade. Among the underachieving children, the percentages were similar in those with and without substantial plaque in the 6–7 years age group but greater in those with substantial plaque in the 10–11 years old. Among the underachieved, the percentages were lower in children who had toothache (8.8, 2.6%) compared to those without toothache (10.5 and 4.6%) in age groups 6–7 and 10–11 years respectively. These data had actually meant that there were more children who did not have toothache and had performed poorly; in contrast to the claim. The distribution of subjects did not offer much information to allow a reasonable interpretation. The *p*-values from the chi-squared tests only indicated significant associations between plaque accumulation and toothache and, school performance but do not suggest an estimate of effect and direction of the relationship to help interpretation. Hence, it was unjustified to claim that poor oral health condition impacted on school performance when the data did not support it.

We believed that those results have been misinterpreted and inaccurately concluded. The error may have originated from an unclear objective of analysis and inappropriate choice of statistical method. To understand the results more effectively, we had re-examined the figures in Table 3 [1] and presented our results. We set our aim to investigate the impact of poor oral health on school performance and hypothesized that children are more likely perform poorly at school if they had oral health problem. The objective of statistical analysis was to compare the odds of having poor mathematics grade between children who had substantial plaque accumulation / toothache and those without substantial plaque / toothache using odds ratio analysis. Plaque and toothache were the exposure variable and school grade was an outcome variable. The next step was to define a ‘poor grade’. Previous studies on similar topic had collapsed ordinal outcomes and contrasted between ‘average/below-average/poor’ and ‘above-average/excellent’ and, between ‘fair/poor’ and ‘good/very-good/excellent’ school grades [2, 3]. Collapsing the classes has to be done carefully to avoid misclassification issues which can affect interpretation later. Different grades that represents contrasting values or norms should be avoided. In this report, two definitions of poor grade were analysed and presented; the *Underachieving* was contrasted to fair/good/excellent grades and; the *Underachieving/fair,* to good/excellent.

We used the data in Table 3 [1], maintained separate analysis for toothache and plaque accumulation and, by age groups 6–7 and 10–11 years-old. As both the outcome and exposure variables were binary, the data could be analysed using a simple chi-square test or using odd ratio analysis. We selected the latter with exact method because it provides both the estimate and direction of the effect of exposure, and facilitates interpretation of the results compared to the former. Significant level was set at 5% and analysis was carried out using STATA/IC 15.1.

Results showed that the odds ratios for the effect of toothache were not significant in both age groups and for both definitions of poor performance (Tables 1 and 2). For plaque accumulation, the odds of having underachieving/fair grades was statistically significantly twice greater (OR = 2.0, 95%CI: 1.16, 3.48) in children aged 10–11 years with substantial plaque (Table 3). No other result was significant (Table 4). At this point, the hypothesis that claims that toothache impacted on school performance was rejected but there was plausible evidence to suggest for the impact of substantial plaque.

**Table 1:** Odds ratio (OR) for the association between toothache and poor (underachieve) school performance in age groups 6–7 and 10–11 years-old

Toothache	Underachieve n (%)	Fair/Good/Excellent n (%)	Total	OR (95% CI)	p
Age 6–7					
Yes	19 (8.8)	196 (91.2)	215	0.83 (0.41, 1.65)	0.6
No	23 (10.5)	197 (89.6)	220	1	
Age 10–11					
Yes	4 (2.6)	151 (97.4)	155	0.56 (0.12, 2.25)	0.4
No	7 (4.6)	147 (95.5)	154	1

**Table 2:** Odds ratio (OR) for the association between toothache and poor (underachieve/fair) school performance in age groups 6–7 and 10–11 years-old

Toothache	Underachieve/Fair n (%)	Good/Excellent n (%)	Total	OR (95% CI)	p
Age 6–7					
Yes	53 (24.6)	162 (75.4)	215	1.4 (0.88, 2.33)	0.1
No	41 (18.6)	179 (81.4)	220	1	
Age 10–11					
Yes	22 (14.2)	133 (85.8)	155	1.0 (0.52, 2.11)	0.9
No	21 (13.6)	133 (86.4)	154	1

**Table 3:** Odds ratio (OR) for the association between plaque accumulation and poor (underachieve/fair) school performance in age groups 6–7 and 10–11 years-old

Substantial plaque	Underachieve/Fair n (%)	Good/Excellent n (%)	Total	OR (95% CI)	p
Age 6–7					
Yes	58 (24.9)	175 (75.1)	233	1.5 (0.95, 2.40)	0.07
No	44 (18.0)	200 (82.0)	244	1	
Age 10–11					
Yes	32 (21.1)	120 (79.0)	233	2.0 (1.11, 3.64)	0.01*
No	28 (11.7)	211 (88.3)	244	1

**Table 4:** Odds ratio (OR) for the association between plaque accumulation and poor (underachieve) school performance in age groups 6–7 and 10–11 years-old

Substantial plaque	Underachieve n (%)	Fair/Good/Excellent n (%)	Total	OR (95% CI)	p
Age 6–7					
Yes	23 (9.9)	210 (90.1)	233	1.0 (0.52, 1.92)	> 0.9
No	24 (9.8)	220 (90.2)	244	1	
Age 10–11					
Yes	10 (6.6)	142 (93.4)	152	1.6 (0.59, 4.43)	0.3
No	10 (4.2)	229 (95.8)	239	1	

## Discussion

Dental journals has been criticized for their lack of quality evidence [4]. These were attributed to the limitation in the study design, statistical methods used and interpretation of the results from analysis. In Maharani et al. it was the latter two. This article was set out to demonstrate that the conclusion about the association between poor oral health and school performance in Maharani et al. [1] was not supported by their data due to inappropriate choice of statistical method and error in interpreting the result and; an alternative analysis was proposed. A step by step approach to analysis was presented by stating the objectives, refining the definition of the exposure and outcome before selecting an optimum analysis tool. The analysis objective statement described the parameters to be compared (odds), the outcome variable (grades), comparison group (e.g. toothache vs no toothache) and statistical test to be used (odds ratio analysis). Refining an outcome variable is very important and it may involve collapsing the original classification to discriminate the group to be studied. The definitions for poor performance in the present report had allowed us to reasonably state, for example, ‘there was a statistically significant evidence that children in the study with substantial plaque had two times greater odds to have underachieving/fair grades in mathematics’. This statement comprises an estimate of effect and a clear direction of impact; which is much better than an association statement.

However, there were also limitations when the underachieving/fair grades were defined as poor performance. The significant impact would only be true if a fair grade with marks 55–64 is considered as poor school performance in real practice but that is not common. The mark is considered as a pass in most education system, thus making that combination impractical. There could also be an implication when applying the result in prevention activities. A large number of children will need to be treated to make a small improvement in the grades and as a result the challenge to improve the grades from fair to good/excellent from oral health activities alone would be monumental. On the contrary, if a fair grade was not considered as poor performance, the association between substantial plaque and school performance diminished (see Table 4), suggesting that fair a grade contributed to an arbitrary effect to the significant result. Further, comparison of results between studies would be more complicated when definitions are inconsistent. These further emphasize the importance of specifying a reasonable and practical definition for the outcome and exposure. Based on these reasons, it may not be justified to consider underachieving/fair as poor performance; hence the data in that study has no evidence to support the hypothesis.

This report had used odds ratio analysis which offers an estimate for, and direction of the effect compared to result from a chi-squared test. The method also requires a clear definition of exposure and outcome variables and, their classifications before an analysis can be performed, thus providing an easier interpretation of result that is consistent with the hypothesis. Although the significant finding in the present analysis was consistent with Maharani et al. for the age respective group, the interpretation of the latter was dubious, i.e. it was not clear how a greater percentage of underachieved and, lower percentage of excellently graded children had contributed to the significant *p*-value. Isolating the poor performance group and contrasting them to the others would allow the analysis to focus on the subject of interest and facilitates interpretation of the findings. Odds ratio analysis was chosen for these advantages over the chi-square tests; which would have given similar results but without the estimate and direction for the association.

## Conclusion

It was concluded that the data in Maharani et al. [1] do not support the claim for the relationship between poor school performance and oral health as reported. It is proposed that researchers should set a clear objective on what they want to achieve in their analysis to avoid misinterpretation of the results and to consult a statistician for better understanding of the analysis and results. The error in Maharani et al., may not be intentional but may have supported earlier claims about the limitation of evidence in dental research because of poor methodology [4].

## Data Availability

All data is contained within this and the previous manuscript.

## References

[1] Maharani DA, Adiatman M, Rahardjo A, Burnside G, Pine C. An assessment of the impacts of child oral health in Indonesia and associations with self-esteem, school performance and perceived employability. BMC Oral Health [Article]. 2017;17(1):65. 10.1186/s12903-017-0358-5 PMC5361816 28327110

[2] Paula JS, Lisboa CM, de Castro Meneghim M, Pereira AC, Ambrosano GMB, Mialhe FL (2016). School performance and oral health conditions: analysis of the impact mediated by socio-economic factors. International Journal of Paediatric Dentistry [Article].

[3] Blumenshine SL, Vann WF, Gizlice Z, Lee JY (2008). Children's school performance: impact of general and oral health. Journal of Public Health Dentistry [Article].

[4] Lesaffre EJJ, Jocelyne F, Leroux B, Declerck D. Statistical and Methodological Aspects of Oral Health Research. Chichester: Wiley; 2009.

