# Supplementary material for: Is there evidence for the impact of poor oral health on school performance?
Source: BMC Oral Health. 2019 Jul 11;19:143. doi: 10.1186/s12903-019-0822-5 (PMC6625084; doi:10.1186/s12903-019-0822-5)
Supplement: Related texts

# Reply: Response to “Is there evidence for the impact of poor oral health on school performance?”

**Authors:** Girvan Burnside, Diah Ayu Maharani, Cynthia Pine

**Affiliations:** 3 0000 0004 1936 8470 grid.10025.36 Department of Biostatistics, University of Liverpool, Liverpool, England UK; 4 0000000120191471 grid.9581.5 Department of Preventive and Public Health Dentistry, Faculty of Dentistry, Universitas Indonesia, Jakarta, Indonesia; 5 0000 0001 2171 1133 grid.4868.2 Dental Public Health, Barts and The London Institute of Dentistry, Queen Mary University of London, Whitechapel, London, England UK

## Response

The authors of the communication suggest that the results are misinterpreted based on an unclear objective and misclassification of the school performance variable. The objective was to investigate whether there was any association between school performance and caries. The particular analysis discussed considered the association between school performance classified by the teacher into four categories (underachieving, fair, good, excellent) and reported toothache in the last 12 months. We chose to carry out a simple association analysis using a chi-squared test, without collapsing the categories. This choice was made prior to the analysis being carried out.

The re-analysis shows that by collapsing the categories into underachieving/fair vs good/excellent, or underachieving vs fair/good/excellent, there is no significant association. However, if the authors had chosen to collapse into underachieving/fair/good vs excellent, there would be a significant association.

We acknowledge that an a priori binary classification of the school performance variable would have made the results of this analysis easier to interpret. However, given that we chose to keep the variable in the four categories supplied by the teacher, we do not feel that it is incorrect to state that there is evidence of an association between school performance and reported toothache. We also acknowledge that we could have been clearer in our interpretation that the difference between those with and without toothache appears to lie in the proportion of students classified as excellent.
